# Multimodal In Vivo Imaging of Retinal and Choroidal Vascular Occlusion

**DOI:** 10.3390/photonics9030201

**Published:** 2022-03-21

**Authors:** Van Phuc Nguyen, Tianye Zhu, Jessica Henry, Wei Zhang, Xueding Wang, Yannis M. Paulus

**Affiliations:** 1 Department of Ophthalmology and Visual Sciences, University of Michigan, Ann Arbor, MI 48105, USA;; 2 Department of Biomedical Engineering, University of Michigan, Ann Arbor, MI 48105, USA;

**Keywords:** photoacoustic microscopy, PAM, optical coherence tomography, OCT, retinal vein occlusion, choroidal vascular occlusion, multimodal retinal imaging

## Abstract

Photoacoustic microscopy (PAM) is an emerging retinal imaging technique that can provide high spatial resolution and high contrast of chorioretinal vessels. PAM is compatible with optical coherence tomography (OCT) and fluorescence imaging, allowing for development of a multimodal imaging system that combines these imaging modalities into one. This study presents a non-invasive, label-free in vivo imaging of retinal and choroidal vascular occlusion using multimodal imaging system, including PAM and OCT. Both retinal vein occlusion (RVO) and choroidal vascular occlusion (CVO) were clearly identified selectively using a spectroscopic PAM imaging. RVO and CVO were created in six rabbits using laser photocoagulation. The dynamic changes of retinal vasculature were observed and evaluated using color fundus photography, fluorescein angiography, OCT, and PAM. The position of RVO and CVO were imaged with different wavelengths ranging from 532 to 600 nm. The data shows that occluded vessels were clearly distinguished from the surrounding retinal vessels on the PAM images. This advanced imaging system is a promising technique for imaging retinal ischemia in preclinical disease models.

## 1. Introduction

Retinal vein occlusion (RVO) has been demonstrated to be a frequent source of vision loss in elderly patients [1]. It is the second most common retinal vascular disease, only behind diabetic retinopathy [2]. RVO affects up to 16 million people worldwide and has a prevalence between 1 and 2% in people over 40 years old [3,4]. Retinal vein occlusion can present as either branch retinal vein occlusion (BRVO) or central retinal vein occlusion (CRVO) [5]. Another ischemic pathology occurs in the choroidal layer, named choroidal vascular occlusion (CVO) or choroidal ischemia, which causes serious retinal disorders due to disruption the blood flow to the choroid of the eye [6–8]. In human patients, CVO may occur from several etiologies, including ocular compression [9–11].

It is imperative to track progression of neovascularization caused by RVO or CVO, and current imaging techniques have various advantages and limitations. Developing an appropriate evaluation method is essential for quantitatively comparing pre- and post-treatment and overall disease progression. The most common imaging technologies for neovascularization include fundus fluorescein angiography (FA) and indocyanine green angiography (ICGA). FA is widely used due to its ability to diagnose complications including nonperfusion and neovascularization [12]. FA, however, has the limitations of being invasive, limited in scope, and has the potential for adverse side effects [13,14]. Indocyanine green (ICG) dye is water-soluble with an absorption peak between 800 and 810 nm [15]. Due to its tendency to rapidly bind to plasma protein, the contrast does not readily leak from choroidal vasculature to allow for choroidal imaging [16]. ICG and FA, however, lack 3D depth information [17–19].

OCT and OCT angiography (OCTA) are also frequently utilized. OCT non-invasively produces cross-sectional retinal images. This data can be used to diagnose structural changes such as macular edema and neovascularization [20–22]. However, B-scan OCT images allow limited evaluation of the choroid and choriocapillaris vasculature [23]. Additionally, the images are limited in their evaluation of newly formed vasculature. OCTA is three-dimensional, non-invasive, and can demonstrate microvascular structures of neovascularization [24,25]. However, the modality cannot demonstrate leakage and is unable to visualize microaneurysms and choroidal microvasculature [26].

Photoacoustic microscopy (PAM) is a potential solution to these limitations and can be integrated with OCT. PA imaging is non-invasive and non-ionizing, with a high resolution and depth of penetration [27–29]. The images are produced when acoustic waves are generated by absorption of short, pulsed laser light in chromophores like hemoglobin, melanin, and lipids [17]. Our group has previously demonstrated multimodal OCT and PAM imaging that can differentiate retinal and choroidal vessels in vivo with high contrast and resolution [30–32]. The custom system provides a lateral resolution of 3.8 μm for OCT and 4.1 μm for PAM, respectively, achieving contrast to distinguish RNV and RVO. Previous studies have primarily used exogenous contrast agents including nanorods, nanostars, or chain-like gold nanoparticles where the particles specifically targeted the regions of neovascularization [17,19,33,34].

Due to the lack of exogenous contrast agent in this study, it is ideal to integrate several imaging modalities including PAM, OCT, scanning laser ophthalmoscopy (SLO), FA, and ICGA for better visualization of ischemia resulting in new retinal vessels. The combination of these technologies was used to provide in vivo ischemia tracking in rabbits with RVO and CVO generated by laser photocoagulation. RVO was generated through a retinal vein occlusion model with laser illumination after administration of Rose Bengal. CVO was produced through laser illumination of choroidal vessels without Rose Bengal. The imaging primarily focused on the multimodal PAM and OCT imaging system’s ability to monitor progression of neovascularization, nonperfusion, and changes in retinal morphology as a result of RVO and CVO.

## 2. Materials and Methods

### 2.1. Multimodal PAM and OCT Imaging

**Figure 1.**
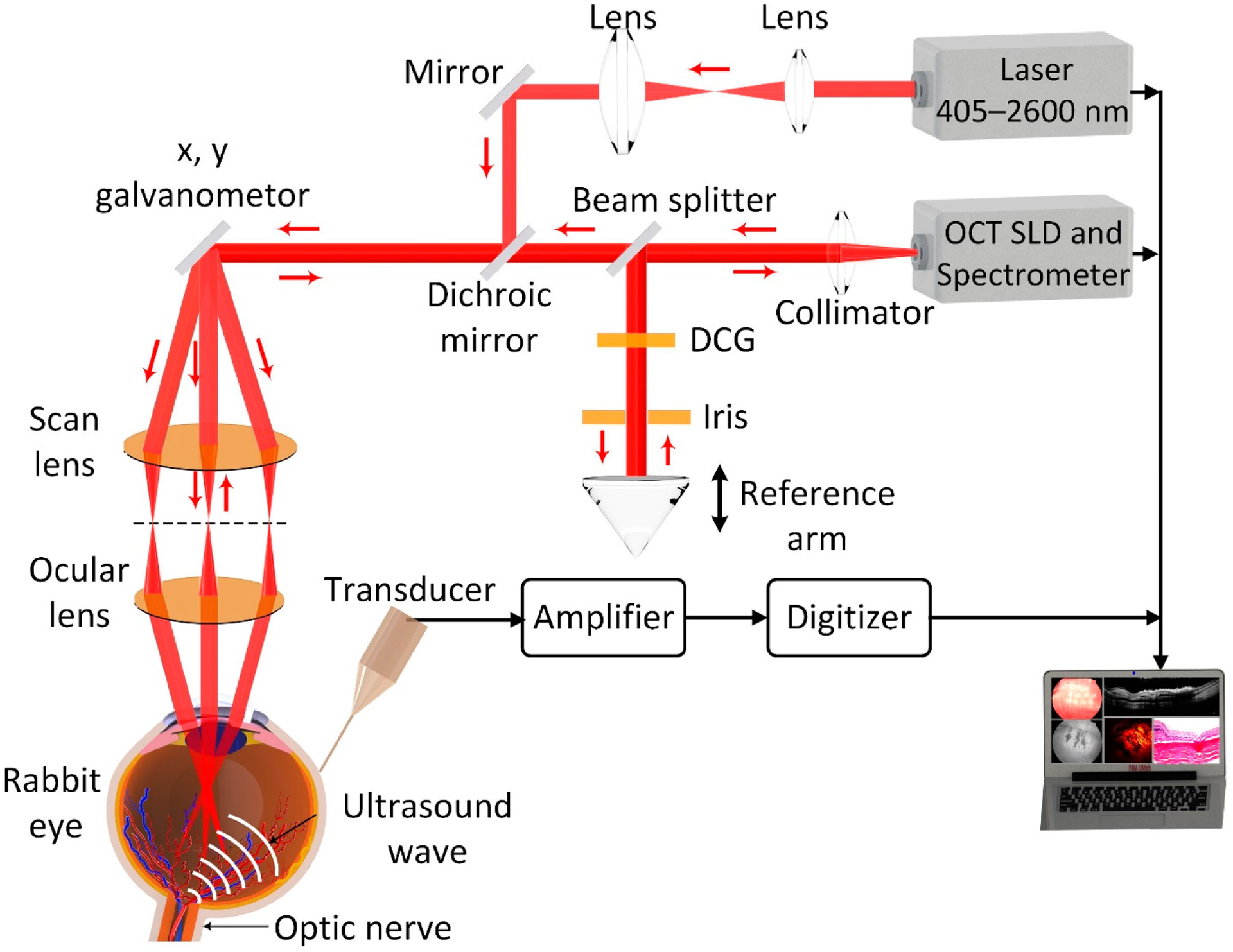
Schematic of the multimodal photoacoustic microscopy and optical coherence tomography imaging system. DCG: dispersion compensation glass. SLD: superluminescent diode.

A custom-made noninvasive and high-resolution multimodal PAM and OCT imaging system [30,31] is presented in Figure 1. To induce PA signal, an OPO laser system with a nanosecond pulse duration of 3–5 ns was used (NT-242, Ekspla, Lithuania). The excitation wavelength can be adjusted from 405–2600 nm with a pulse repetition rate = 1 kHz. The output laser light was filtered and collimated to create a homogeneous circular beam size of approximately 2 mm. By passing through the cornea and lens of the eye, the laser was focused on the fundus with an estimated diameter of 20 μm. To achieve maximized PA signal, a wavelength of 578 nm with energy of ~80 nJ was selected to illuminate the blood vessels. This energy is about half of the American National Standards Institute (ANSI) limit of the maximum permissible single laser pulse energy on the retina (i.e., 160 nJ at 578 nm and 650 nm) [30,35], helping to avoid thermoacoustic damage which may affect retinal’s function [36,37]. The laser-induced PA signal was detected by a needle transducer with a center frequency of 27 MHz (two-way bandwidth *−*60%; Optosonic Inc., Arcadia, CA, USA). The PA signal was then amplified using a 1.4 dB preamplifier (AU-1647, L3 Narda-MITEQ, NY, USA) and digitized using a 200 MHz (PX1500–4, Signatec Inc., Newport Beach, CA, USA). Both 2D and 3D PAM images were rendered from the recorded data. The PAM system has a lateral and axial resolutions of 4.1 μm and 37.0 μm, respectively. The acquisition time is about 65 s with a resolution of 256 × 256 pixels.

OCT images were obtained by a spectral domain OCT system (Ganymede-II-HR, Thorlabs, Newton, NJ, USA) with additional modification as described elsewhere [30,31]. Two superluminescent diodes (SLD) with a center of 846 nm and 932 nm were used to excite the samples. The lateral and axial resolutions were determined to be 3.8 μm and 4.0 μm, respectively. The OCT excitation beam were coaxially aligned with PAM excitation beam for guiding PAM and helping to interpret PAM results. The 2D OCT images can be obtained within 0.103 s with 512 × 1024 A-lines, and the acquisition rate of 36 kHz.

### 2.2. Animal Preparation

Six New Zealand white rabbits (2–4 months, 2.5–3.0 kg) were obtained from the Center for Advanced Models and Translational Sciences and Therapeutics (CAMTraST) at the University of Michigan Medical School. The animals were divided into two groups: retinal vein occlusion (RVO) and choroidal vein occlusion (CVO). RVO was created by Rose Bengal dye-enhanced photochemical thrombosis laser photocoagulation. CVO models were obtained by treated rabbits with laser photocoagulation without administration of Rose Bengal.

The animal vitals, including mucous membrane color, body temperature, heart rate, and respiratory rate, were monitored and documented every 15 min during the experiment and recovery. Rabbits were anesthetized by intramuscular injection of ketamine (40 mg/kg) and xylazine (5 mg/kg). Tropicamide 1% ophthalmic and phenylephrine hydrochloride 2.5% ophthalmic were applied to dilate the pupil. Topical anesthesia in the eye was implemented using a drop of 0.5% topical tetracaine. A water circulating heat blanket was utilized to maintain the animal’s body temperature. All rabbit experiments were employed under the guidelines of the Association for Research in Vision and Ophthalmology (ARVO) Statement on the Use of Laboratory Animals in Ophthalmic and Vision Research after approval by the Institutional Animal Care and Use Committee (IACUC) of the University of Michigan (Protocol PRO000010388, PI Paulus).

#### 2.2.1. Retinal Vein Occlusion (RVO) Model

RVO models were created on rabbit’s retina using the Rose Bengal dye-enhanced photochemical thrombosis [23,38,39]. A 532 nm green light laser mounted on a slit lamp was utilized to illuminate the target retinal vessels (Vitra 532 nm, Quantel Medical, Cournon d’Auvergne, France). A contact lens (Volk H-R Wide Field, laser spot 2× magnification, Volk Optical Inc., Mentor, OH, USA) was coupled on the cornea to determine the treatment position. Afterwards, Rose Bengal, at a concentration of 50 mg/mL, was intravenously injected into the rabbit via marginal ear vein. 5–10 s after the injection, laser light at a power of 150 mW, aerial spot diameter of 75 μm, and pulse duration of 500 ms were illuminated into the retinal veins. Twenty spots were illuminated at the same position. The treated location was selected at a distance of one-half to one disc diameter from the optic nerve to avoid side effects to the optic nerve such as optic neuropathy [40]. Then, laser power was increased to 300 mW and illuminated for a further 20 shots to avoid immediate reperfusion of the vein. The targeted location on the veins was carefully determined to minimize affecting the adjacent arteries [41].

#### 2.2.2. Choroidal Vascular Occlusion (CVO) Model

The choroidal vascular occlusion (CVO) model was created using laser photocoagulation. Three New Zealand white rabbits were illuminated with a green light laser (Vitra 532 nm, Quantel Medical, Cournon d’Auvergne, France) connected with a Zeiss SL 130 slit lamp (Carl Zeiss Meditec, Jena, Germany). A contact lens (Volk H-R Wide Field, laser spot 2× magnification, Volk Optical Inc., Mentor, OH, USA) was placed on the cornea of the rabbit eye and the target vessels were illuminated with laser at a power of 450 mW with a spot size approximately of 300 μm in aerial diameter, and the pulse duration of 500 ms. Twelve shots of the laser were illuminated into the eye at different positions.

### 2.3. Imaging of RVO and CVO

#### 2.3.1. Color Fundus Photography, FA, ICGA

Before and after laser photocoagulation, all the treated rabbits were monitored with color fundus photography, FA, ICGA, PAM, and OCT for up to 28 days. The color fundus photography, FA, and ICGA images were obtained by the Topcon 50EX system (Topcon Corporation, Tokyo, Japan). Morphology of retinal blood vessels was first imaged with the color fundus photography to evaluate the change of choroidal vessels as well as to quantify blood perfusion within the vessels. Sequentially, FA and ICGA were acquired to examine the vascular occlusion. To acquired FA images, 10% fluorescein sodium at a dose of 0.2 mL (Akorn, Lake Forest, IL, USA) was intravenously administrated in the rabbit marginal ear vein. Sequentially, FA images were acquired immediately after injection. Late-phase FA images were obtained every minute over a period of 15 min. ICGA images were implemented by intravenous injection of 0.2 mL ICG at concentration of 2.5 mg/mL (Patheon Italia S.p.A., MB, Italy).

#### 2.3.2. In Vivo PAM and OCT of RVO and CVO

Retinal blood vessels before and after laser photocoagulation were imaged by PAM and OCT imaging to determine the degree of ischemia and dynamic change of retinal blood vessels. To obtain 2D and 3D PAM and OCT images, rabbits under anesthesia were positioned on two separated stabilization platforms to minimize motion artifacts. The target vessels were selected from the fundus camera integrated on the OCT system, allowing the acquisition of the image at the appropriate location over time. 2D OCT images were acquired with a resolution of 512 × 1024 A-lines. Then, PAM images were acquired using multiple optical wavelengths ranging from 532–600 nm. To acquire emission PA signals, the ultrasound transducer was placed in contact with the conjunctiva of the rabbit eye without applying any pressure to allow the transducer to move freely. Both RVO and CVO were monitored for up to 28 days to evaluate the dynamic change of retinal and choroidal vessels. By raster scanning along two x− and y− axes, 3D volumetric PAM image was achieved and reconstructed using Amira software (Amira 6.0, Fisher Scientific Inc., Hampton, NH, USA). Quantitively measurements such as retinal thickness, laser injury depth, and PA signals were measured by ImageJ software (NIH, Bethesda, MD, USA). The measurement was performed on 2D OCT B scan images. Twelve different positions were selected along the OCT images and average data and standard deviation was determined at each time point.

### 2.4. Histological Analysis

Histological analysis was implemented to evaluate the degree of RVO, CVO, and retinal damage. The rabbits were euthanized at day 28 post-photocoagulation by intravenous injection of euthanasia solution (Beuthanasia-D, 0.22 mg/kg, 50 mg/mL, VetOne, Boise, ID, USA). The eyeballs were sectioned and fixed in Davidson’s fixative solution for 24 h. 100 μL of 4% formaldehyde buffer solution was intravitreally injected into the retina to prevent retinal detachment. The fixed sample was then cut into 5 mm specimens and embedded in paraffin. The embedded tissue was sectioned at a thickness of 4 μm using a Leica autostainer XL (Leica Biosystems, Nussloch, Germany) and stained with hematoxylin and eosin (H&E). The H&E slides were examined under microscope (Leica DM600 light microscope (Leica Biosystems, Nussloch, Germany) and the H&E images were captured using the BF450C camera.

## 3. Results

### 3.1. Retinal Vein Occlusion and Choroidal Vascular Occlusion Cause Severe Retinal Vessel Changes and Increase Vascular Tortuosity

**Figure 2.**
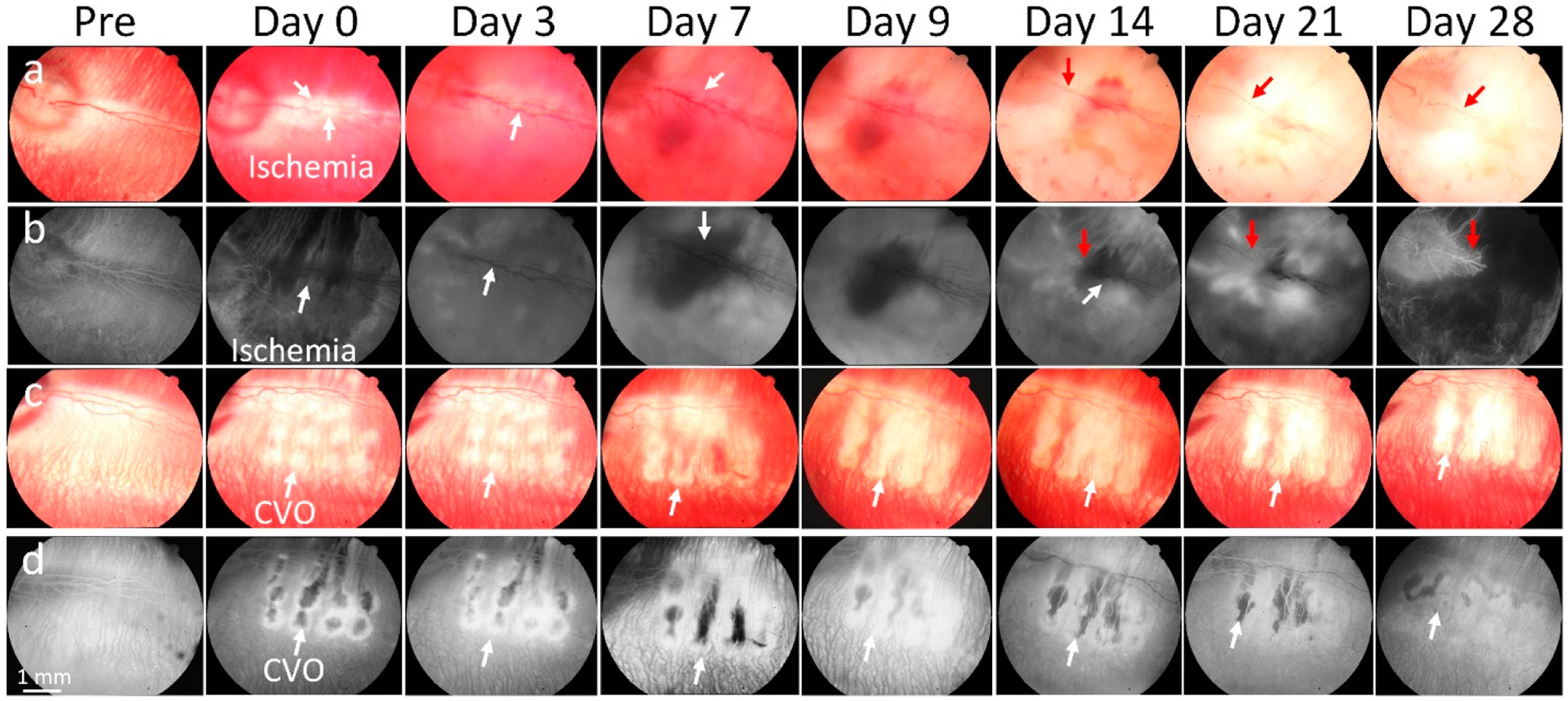
In vivo imaging of retinal and choroidal vascular occlusion: (**a**) color fundus photography, (**b**) fluorescein angiography (FA) imaging of retinal vein occlusion (RVO). (**c**,**d**) Color and FA images of choroidal vascular occlusion (CVO). Images of RVO and CVO acquired pre- and post-laser treatment at different time points: Day 0, 1, 3, 7, 14, 21, and 28. White arrows indicate the targeted treatment areas. Color intensity (color fundus) or fluorescent intensities (FA) reduced post-treatment, confirming the location of RVO and CVO. Hemorrhage appeared at day 7 and 9 post-laser treatment. Red arrows show the new retinal vessels created from day 14.

Retinal vascular occlusion was implemented on the major retinal veins using laser photocoagulation. The location of the occluded vessels in rabbits were confirmed by FA immediately after the laser treatment (Figure 2). The occlusion of retinal vein was observed in all RVO rabbits post-laser illumination. After laser treatment, the location of the occluded vessels was clearly observed on fundus images due to the change of color intensity at the treated area (Figure 2a). The blood flow in the retinal vessel was significantly changed. At the upstream from the occlusion position, the retinal vessels became dilated and blood flow redirected to collateral circulation as shown in the fluorescein angiography (FA; Figure 2b). Vascular tortuosity was observed shortly after the vessels were occluded, which is a common event with RVO. Some studies have reported that the occluded vein in mice models spontaneously restored blood flow around 1 week post-laser treatment [42]. Similar to those studies, we found that the occluded vein in RVO rabbits reperfused within 5–7 days post-treatment when the major vein or artery occluded. In this case both major artery and veins were occluded, and retinal vessels were disrupted in this region without evidence of blood perfusion for at least 28 days (Figure 2b). In the CVO model, blood flow at the location of laser site was completely stopped without reperfusion (Figure 2c,d). Laser scars appeared around the laser injury sites, as demonstrated by increased fluorescence observed on ICGA. There was no evidence of the choroidal vasculature changing around the laser site. No evidence of neovascularization was observed on the fundus and FA images.

**Figure 3.**
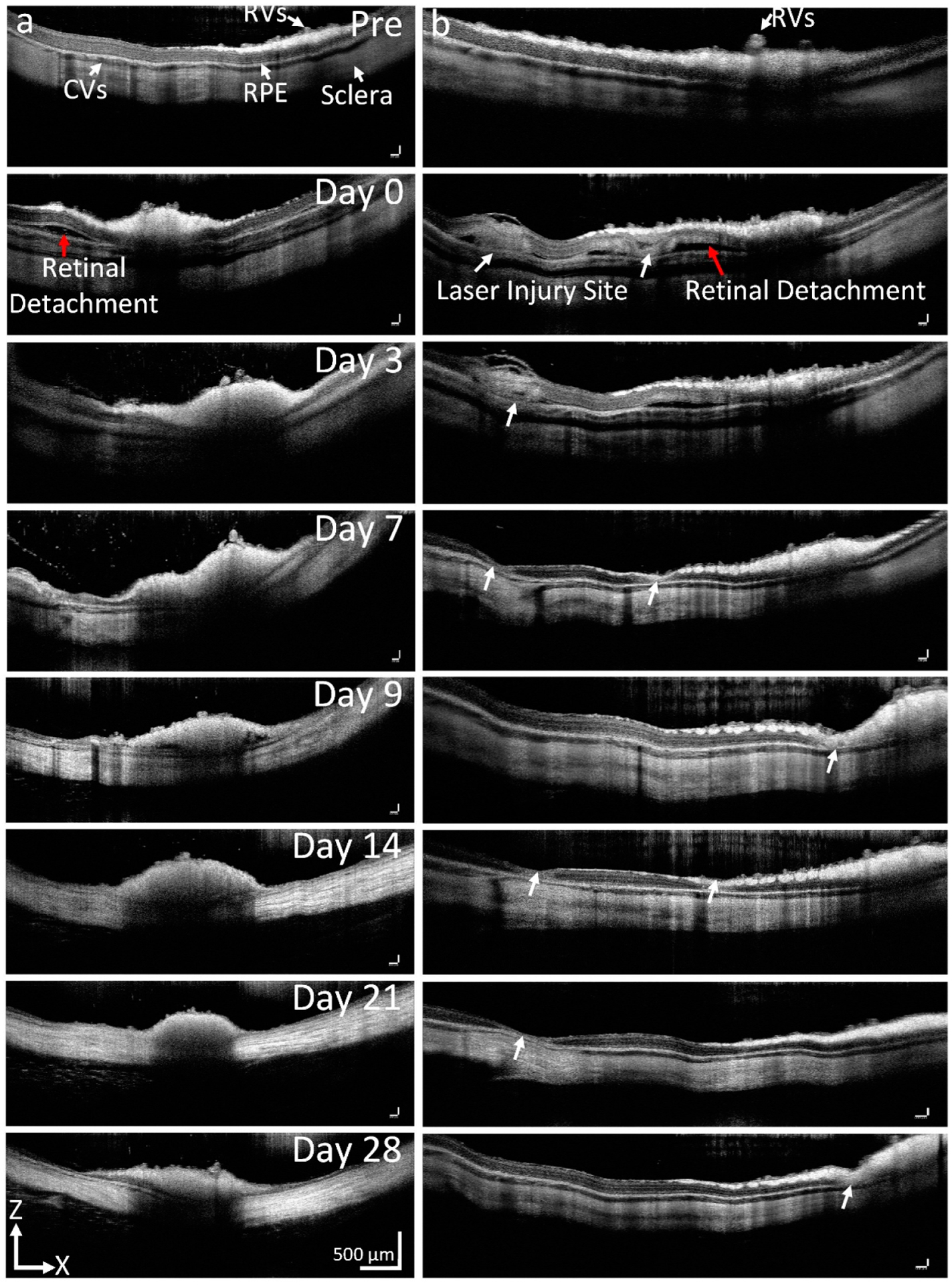
In vivo OCT imaging of RVO and CVO. (**a**,**b**) 2D OCT images acquired along the occluded retinal and choroidal vessels, respectively. Pre-laser treatment, retinal vessels (RVs), choroidal vessels (CVs), ganglion cell layer (GCL), retinal pigment epithelium (RPE), and sclera were clearly observed, and these layers are intact pre-laser. After laser illumination, retinal detachment was observed on both RVO and CVO (red arrows). White arrows show the area of retina affected by laser. No vascular recanalization was observed in both RVO and CVO rabbits.

Both RVO and CVO-induced retinal tissue damage was clearly visualized on 2D OCT images. By acquiring the OCT images along the laser injury sites, we found that localized retinal detachment was observed around the laser injury sites acutely (Figure 3). Retinal detachment was observed on day 0 post-laser treatment and completely resolved at day 3. We observed the changes in retinal thickness and found that the average thickness increased 177.2% from 346.91 ± 19.51 μm at day 0 post-treatment when compared to that of before RVO (thickness = 195.76 ± 15.19 μm for Pre-RVO) due to increased permeability of the occluded vein. Similarly, the retinal thickness was also increased 153.7% in CVO models (thickness = 268.55 ± 12.66 μm at pre vs. 412.85 ± 24.20 μm at day 0). Then, the retinal thickness was significantly reduced to 14% (thickness = 27.72 ± 3.60 μm) for RVO and 11.6% (thickness = 31.25 ± 6.65 μm) for CVO at day 28 due to the loss of ganglion cell layer (GCL), inner plexiform layer, inner nuclear layer, outer plexiform layer, outer nuclear layer, photoreceptors, and retinal pigment epithelium (RPE) layers.

### 3.2. In Vivo PAM Visualization of Ischemia in RVO and CVO

**Figure 4.**
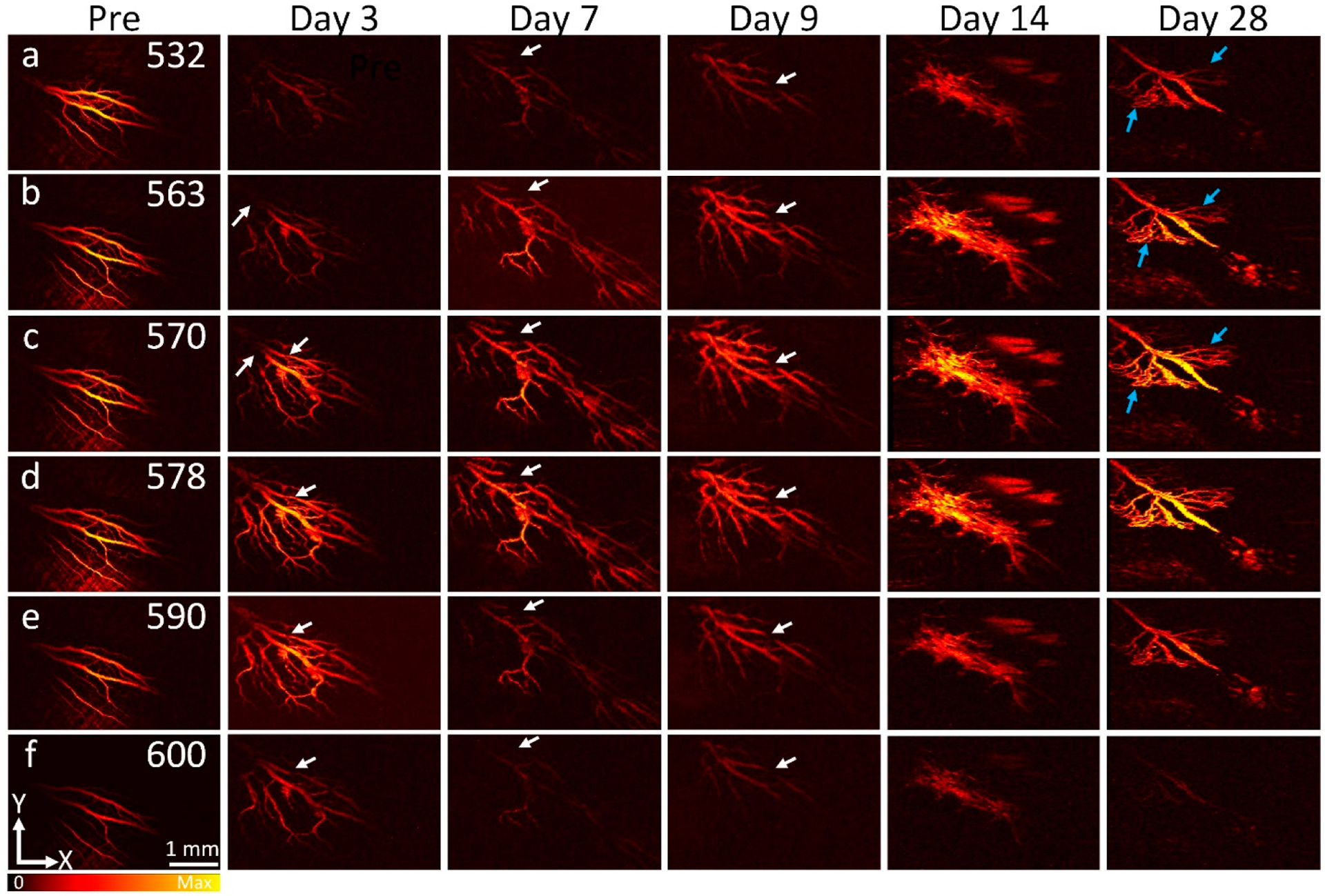
Longitudinal spectroscopic PAM visualization of RVO before and after laser irradiation. (**a**–**f**) PAM images obtained at different wavelengths (532–600 nm) and different time points before and after laser treatment for up to 28 days. White arrows demonstrate the injury vessels. At the treated location, the PAM image contrast was decreased and blurred in comparison to the untreated areas. The treated retinal vessels were detected at all wavelengths. However, the strongest PA contrast occurred at the wavelengths from 563 to 578 nm.

**Figure 5.**
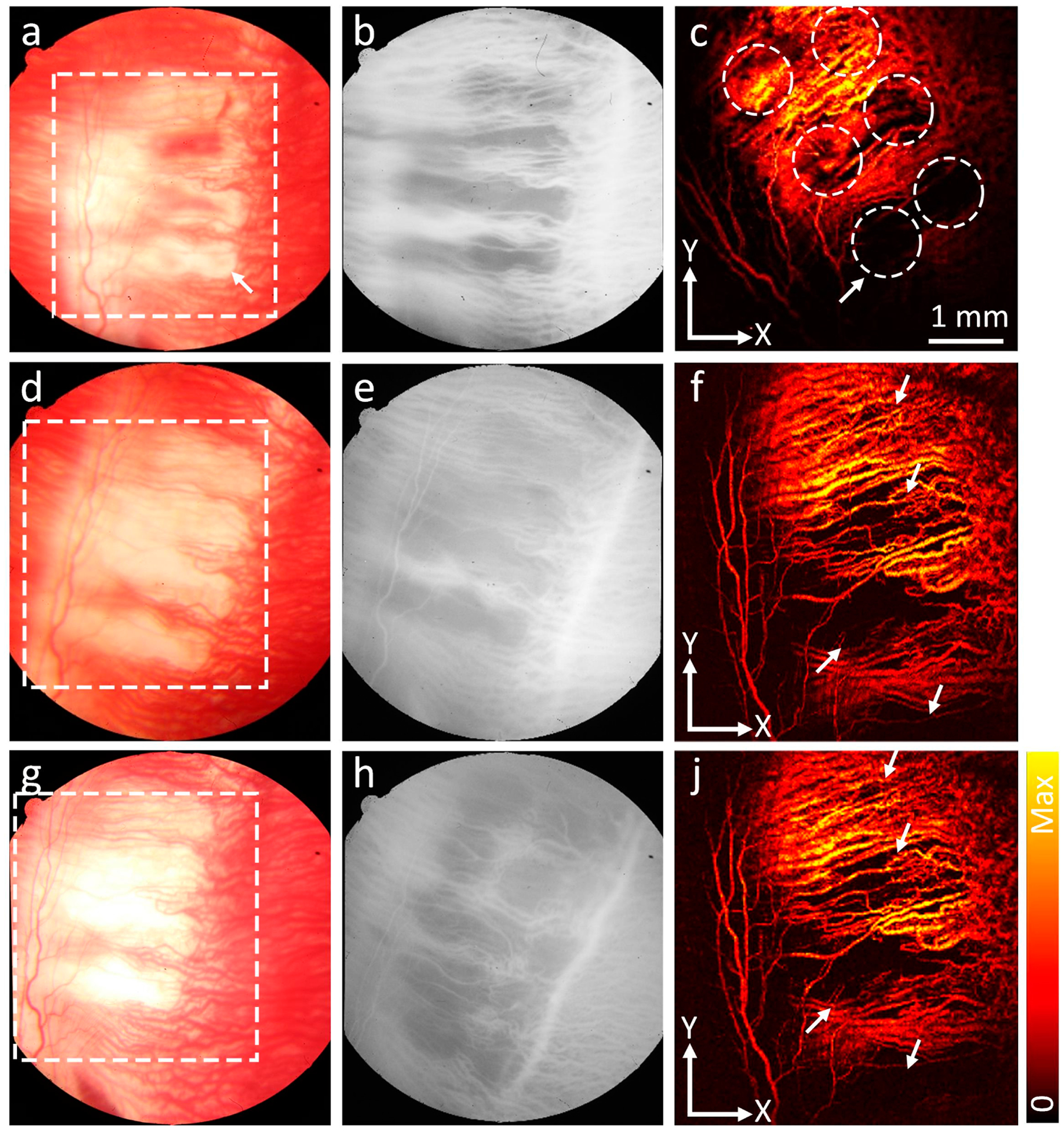
In vivo PAM images of CVO model. Position of CVO was clearly detected on the PAM images obtained at 578 nm. The treated area significantly changed over time. (**a**,**d**,**g**) Color fundus photographs of CVO at day 0, 7,and 28, respectively. (**b**,**e**,**h**) Fluorescein angiography (FA) images of CVO at day 0, 7,and 28, respectively. (**c**,**f**,**j**) Corresponding PAM images obtained at 578 nm along the selected area (white dotted rectangular) shown in a,d, and g at day 0, 7,and 28, respectively. White arrows demonstrate the location of laser injury sites.

In vivo PAM imaging of ischemia was implemented on RVO and CVO rabbits using a custom-built PAM imaging system. After laser treatment, RVO and CVO models were imaged using PAM at different wavelengths from 532–600 nm. This allows for selective detection of RVO and CVO sites. The PAM images before laser treatment show normal morphology blood vessels with high contrast (Figure 4). Note that PAM images obtained at 578 nm show higher contrast compared to that of slower or longer wavelengths due to strong absorption of hemoglobin within the blood vessels (i.e., μ_Hb_ = 270.56 cm^−1^ at 578 nm vs (i.e., μ_Hb_ = 77.76, and 17.28 cm^−1^ at 590 and 600 nm, respectively) [43]. After treatment, the PA contrast at the position of laser injury site was reduced compared to that of the adjacent retinal vessels, indicating retinal/vein or choroidal vessels were occluded (Figures 4b–f and 5). The position and margin of the ischemic vessels obtained by PAM were likely the same as those observed on FA images. We found that the vessel anatomy gradually changed over time. At day 28, the occluded retinal veins almost disappeared downstream. Upstream ischemia created new retinal capillaries and established retinal and choroidal neovascularization (blue arrows). However, no neovascularization was observed in the CVO model. PA signals at the location of laser injury site and untreated sites were measured on the same retinal vessels by ImageJ software (NIH, Bethesda, MD, USA). The results show that the PAM signals at the ischemia site reduced 94.04% and 94.77% at day 7 post-RVO and CVO when compared to the control, respectively (PA_Amplitude_ = 10.15 ± 1.48 (a.u.) for RVO, 7.52 ± 2.54 (a.u.) for CVO vs 170.40 ± 0.98 (a.u.) for control retinal vessels and 143.70 ± 5.00 (a.u.) for control choroidal vessels). The reduced PA signal at the ischemia site was likely caused by interrupted blood flow, resulting in the concentration of hemoglobin at this position reducing significantly. In addition, after laser treatment, blood vessels had shrinkage and thus reduced the PA amplitudes. Note that the PA slightly changed at day 7, 14, and 28 on RVO models. This confirms that the retinal vessels are affected significantly over time. On the other hands, the PA signal at CVO injury site gradually increases likely new capillaries regenerated at the treated area. No significant PA signal change was observed at the untreated area on the CVO model.

### 3.3. Histological Analysis

**Figure 6.**
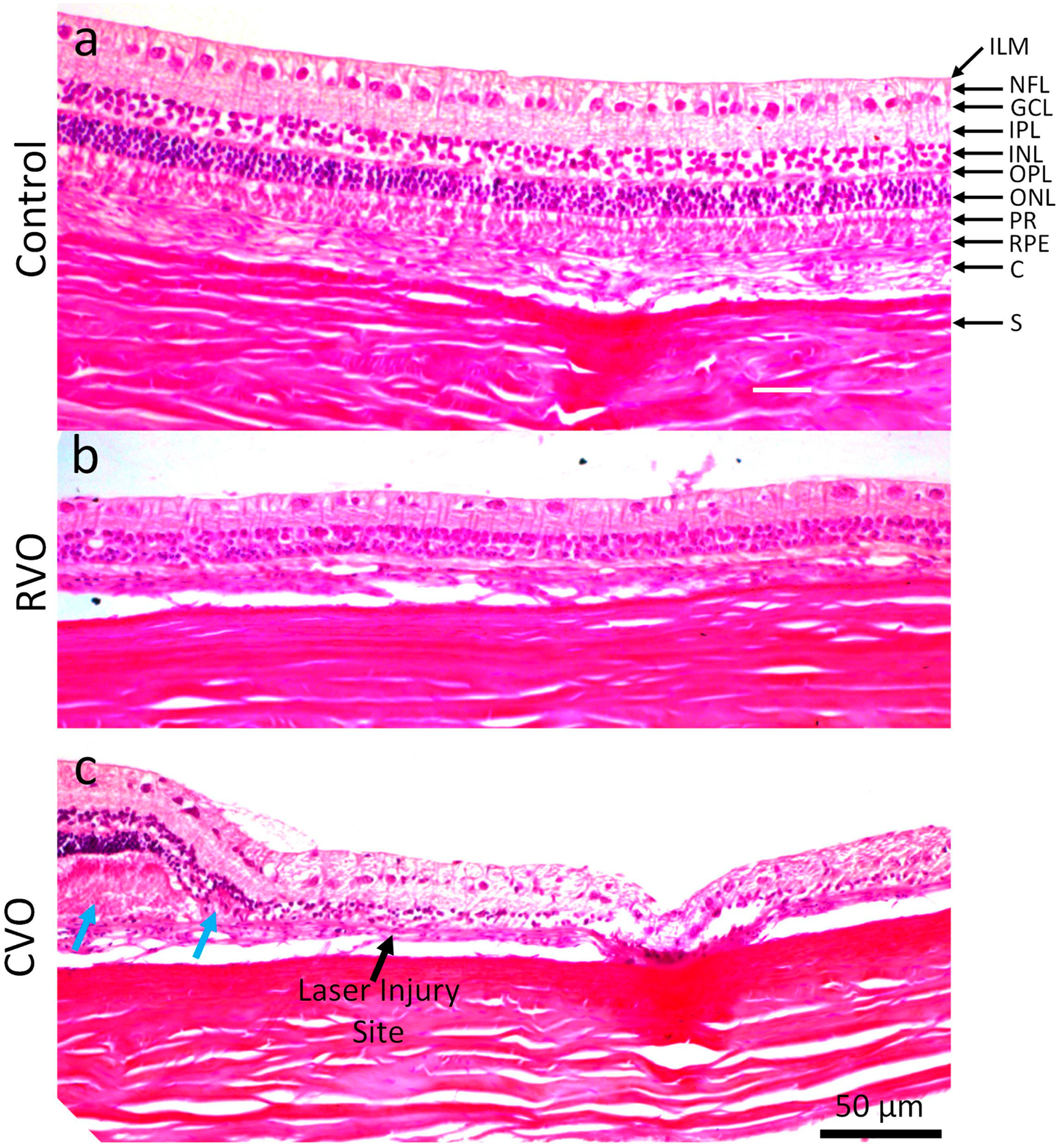
Histopathological analysis of RVO and CVO. (**a**) Hematoxylin and eosin (H&E) images of the retinal tissue without laser treatment. These images clearly show retinal anatomy with different layers. (**b**, H&E images of RVO acquired at day 28 post-laser treatment. (**c**) H&E images of CVO. There were significant changes in the retinal architecture. Blue arrows show the focal area of the outer nuclear, photoreceptor, ganglion cells and inner nuclear layers at the affected region and is associated with loss of adjacent RPE and the morphology of the retina on side of the lesion (black arrow). Note that the retinal thickness was thinner than the control group. Magnification: ×20 and ×40. ILM: inner layer membrane; NFL: nerve fiber layer; GCL: Ganglion cell layer; IPL: Inner plexiform layer; INL: Inner nuclear layer; OPL: Outer plexiform layer; ONL: Outer nuclear layer; PR: Photoreceptors, C: Choroid; S: Sclera.

**Figure 7.**
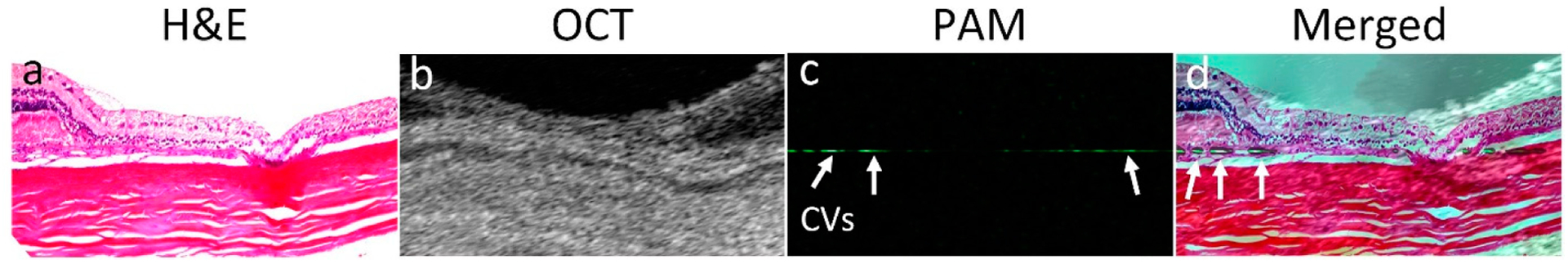
Co-registration of H&E, OCT, and PAM: (**a**) H&E image. (**b**) Cross-sectional OCT image. (**c**) B-scan PAM image. (**d**) Overlay image. White arrows indicate the location of choroidal vessels (CVs).

Hematoxylin and eosin (H&E) histology images of treated and untreated retinal tissues were obtained at day 28 post-treatment. No evidence of anatomic disorganization was observed in the control images (Figure 6). All GCL, inner plexiform layer (IPL), inner nuclear layer (INL), outer plexiform layer (OPL), outer nuclear layer (ONL), photoreceptors, RPE, and choroid layers were clearly visualized with normal architecture (Figure 6a). On the other hand, RPE loss, disorganization of the inner and outer plexiform layers, and photoreceptors were obviously detected on RVO and CVO tissues (Figure 6b,c). Bruch’s membrane disruption (blue arrow), cellular debris, and macrophages were detected (black arrow), indicating the evidence of choroidal scar (Figure 6c). In addition, retinal thickness was significantly reduced both on RVO and CVO models. The retinal thickness was reduced by 58.31% in RVO and 51.39% in CVO (i.e., thickness = 132.96 ± 5.62 μm for control vs. 55.43.43 ± 1.14 μm for RVO and 64.63 ± 5.62 μm for CVO, N = 6; *p* < 0.001). To improve visualization of the CVO, H&E images were co-registered with PAM and OCT as shown in Figure 7. Both OCT and H&E clearly show the location of the injury sites (Figure 7a,b). In contrast, there is minimal PAM signal observed on the B-scan PAM image (Figure 7c) due to the loss of choroidal vessels post-laser illumination. White arrows show the position of choroidal vessels.

## 4. Discussion

This report presents an advanced label free multimodal PAM and OCT imaging technique for precise visualization of retinal and choroidal vascular occlusion in large animals. Both retinal and choroidal ischemia were performed by laser photocoagulation. The targeted vessels were occluded for up to 28 days without reperfusion and confirmed by several imaging modalities, such as color fundus photography, FA, ICGA, PAM, and OCT. We found that retinal ischemia caused several changes on the rabbit retina such as vessels tortuosity, major retinal vessels completely disappeared, and neovascularization was established in RVO model at day 28 post-treatment. On the other hand, choroidal vessel ischemia caused local choroidal vessels damage within the laser spot size, and vascular scars appeared at day 7 post-laser treatment. In patients, retinal ischemia may cause hypoxia, leading to vascular endothelial cell growth factor (VEGF) generation and the development of neovascularization of the iris and retina and macular edema [5]. Several studies have reported that retinal ischemia induced ocular neovascularization in mice [44], rats [43], pigs [45], and in patients [9–11]. However, the hypothesis of ischemia trigger VEGF levels contributing to the observed retinal neovascularization is unclear. Our study found that venous recanalization did not raise neovascularization. Future studies should address defining the relationship between ischemia and retinal neovascularization in experimental RVO, using molecular probes such as HYPOX-4 [46].

An advantage of this study is that the ischemia on the retina was performed in large animals, rabbits, which have an eyeball axial length similar to human size (i.e., axial length = 23 for human vs 18.1 mm for rabbit), allowing one to translate to clinical application. In addition, the ischemia can be visualized in three dimensions using PAM and spectroscopic PAM, allowing identification of the stage and margins of the tissue ischemia selectively and precisely. However, the imaging acquisition time is approximately 65 s, contributing to reducing unwanted motion artifacts that may occur during in vivo experiments and may affect the quality of the image.

## 5. Conclusions

The current study has demonstrated an advanced technique for detection of retinal and choroidal vascular occlusion using high resolution multimodal PAM and OCT imaging. The system allows to detect the location of dynamic change of retinal blood vessels longitudinally with high specificity and sensitivity.
